# Development of a Self-Charging Lithium-Ion Battery Using Perovskite Solar Cells

**DOI:** 10.3390/nano10091705

**Published:** 2020-08-29

**Authors:** Yeongbeom Kim, Hyungkee Seo, Eunbi Kim, Jaekwang Kim, Inseok Seo

**Affiliations:** 1 Research Center for Advanced Materials Development (RCAMD), School of Advanced Materials Engineering, Jeonbuk National University, Baekje-daero 567, Jeonju 54896, Korea; kyb5646@naver.com; 2 School of Chemical Engineering, Jeonbuk National University, Baekje-daero 567, Jeonju 54896, Korea; hkseo@jbnu.ac.kr (H.S.); keb821@naver.com (E.K.); 3 Department of Solar & Energy Engineering, Cheongju University, Cheongju 360-764, Chungbuk, Korea

**Keywords:** self-charging, LiFePO_4_, Li_4_Ti_5_O_12_, lithium-ion batteries, perovskite solar cell

## Abstract

This study demonstrates the use of perovskite solar cells for fabrication of self-charging lithium-ion batteries (LIBs). A LiFePO_4_ (LFP) cathode and Li_4_Ti_5_O_12_ (LTO) anode were used to fabricate a LIB. The surface morphologies of the LiFePO_4_ and Li_4_Ti_5_O_12_ powders were examined using field emission scanning electron microscopy. The structural properties of the two powders were investigated using X-ray diffraction. The electrochemical properties of the LiFePO_4_-Li and Li_4_Ti_5_O_12_-Li half cells and of the full cell were investigated. The LiFePO_4_-Li_4_Ti_5_O_12_ full cell showed an excellent Coulombic efficiency of 99.3% after 100 cycles. CH_3_NH_3_PBI_3_ (MAPbI_3_) perovskite solar cells (PSCs) were fabricated using a spin coating technique. A single PSC showed a power conversion efficiency of 12.95%. In order to develop a self-charging system for LIBs, four single PSCs connected in series were used as an LFP-LTO battery. The integrated PSC system showed a power conversion efficiency of 12.44%. The PSC-LIB coupled device showed excellent overall self-charging conversion and a storage efficiency of 9.25%.

## 1. Introduction

With the rapid depletion of fossil fuels, the global concern over environmental issues has increased and extensive efforts are being invested in the development of novel systems for energy production and storage [1,2,3]. Over the past few decades, a rapid increase in CO_2_ emissions has caused global warming, amplifying the need for renewable energy sources such as wind, solar, and water. Solar energy is an important renewable energy source. However, the intermittent nature of solar energy is a major disadvantage that limits its widespread application. This limitation can be overcome with the use of lithium-ion batteries (LIBs), which can be directly coupled to solar power generators [4,5]. As electronic devices become ever more important, the significance of LIBs as energy storage devices seems to be limitless. One of the large-scale applications of LIBs is in electric vehicles. Electric vehicles are very important from an environmental standpoint as they do not produce CO_2_ emissions. However, though the vehicles themselves do not produce CO_2_, the electricity generated from fossil fuels that is used to charge the vehicles can produce a large amount of CO_2_. Hence, in order to realize the environmental advantages of electric vehicles, it is imperative to charge them with electricity generated from renewable sources. In addition, electric vehicles are inconvenient to use because of a lack of charging stations [6]. If electric vehicles could be charged using photocharging systems, their market could be expanded. Self-charging LIBs charged with renewable energy can play an important role in improving the performance of portable electronic devices. Self-charging LIBs can act as independent energy sources and are beneficial for the continuous operation of sensors. 

Solar cells are promising self-charging systems for LIBs. Among the various types of solar cells, perovskite solar cells (PSCs) [7,8,9] and dye-sensitized solar cells [10,11,12] are considered potential self-charging sources for LIBs. Various studies have been carried out on solar energy storage using capacitors [13,14,15,16,17]. A few studies have been carried out on the use of solar energy as a self-charging source for LIBs. In 2012, Guo et al. fabricated an integrated power pack consisting of a dye-sensitized solar cell and LIB using double-sided TiO_2_ nanotube arrays [18]. Although the LIB charged for 8 min at 3 V and showed a discharge capacity of 33.89 μAh, the overall photoelectric conversion and storage efficiency of the device were very low. In 2016, Mahmoudzadeh et al. demonstrated the use of a dye-sensitized solar cell as a charging system for redox batteries [19]. The redox battery used Li_2_WO_4_ as the anode, LiI as the cathode, and a LISICON film as the separator. The solar cell-LIB coupling device showed a discharge capacity of 240 μAh/cm^2^ and a storage efficiency of 78%. However, the solar cell showed a very low efficiency of only 1.7%. Xu et al. used four PSCs connected in series as the solar energy source for a LFB-LTO LIB [20]. The device showed an overall photoelectric conversion and storage efficiency of 7.8% and an energy storage efficiency of only around 60%. 

In this study, a coupling device consisting of four high-efficiency single PSCs connected in series and an LFP-LTO LIB was developed. The surface morphologies and structural properties of the LFP cathode and LTO anode powders were investigated using field emission scanning electron microscopy (FE-SEM) and X-ray diffraction (XRD), respectively. The electrochemical properties of the LiFePO_4_ and Li_4_Ti_5_O_12_ half cells (with Li) and the full cell were investigated. In order to develop a self-charging LIB, four single series-connected PSCs were coupled with an LFP-LTO battery. 

## 2. Materials and Methods 

### 2.1. Fabrication of the LIB

The cathode was fabricated by blending LFP powder with carbon black (Super P) and polyvinylidene difluoride at a weight ratio of 8:1:1. The anode was prepared in the same manner as the cathode using LTO powder. N-Methyl-2-pyrrolidone was used as the solvent. A mixture of 1.2 M LiPF_6_ in a 1:1 (*v/v*, %) solution of ethylene carbonate and dimethyl carbonate was used as the electrolyte. Fabrication of the electrode sheet was carried out using a micrometer film applicator and a roll pressing process. The LIBs were assembled using CR 2032 coin-type cells and pouch cells in an Ar-filled glove box. The pouch cell was 4 × 4 cm in size. Ni and Al were used as the cathode and anode lead tabs, respectively.

### 2.2. Fabrication of PSCs

**Figure 1:**
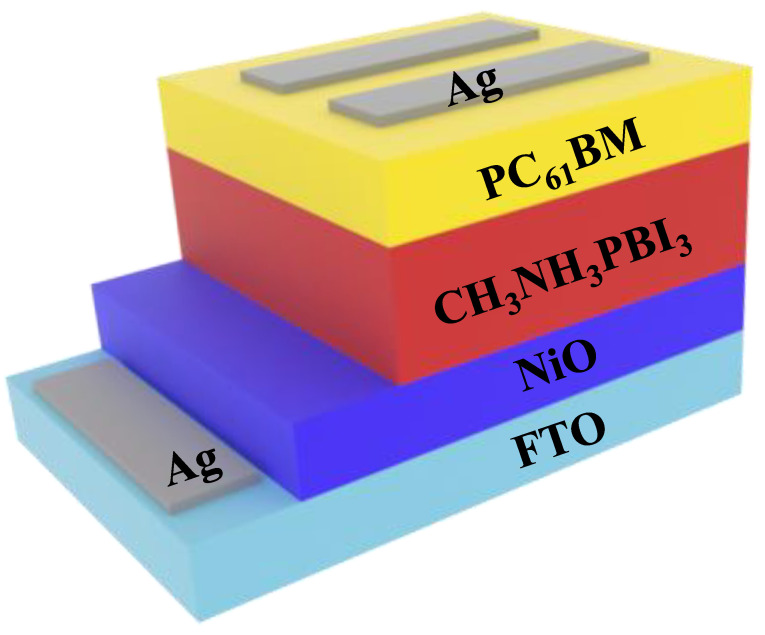
A schematic diagram of the perovskite solar cell (PSC).

CH_3_NH_3_PBI_3_ (MAPbI_3_) perovskite solar cell modules with reasonable performance were fabricated using a spin-coating technique. To construct i-PSC, we adopted a new method: patterning of the fluorine-doped tin oxide (FTO) substrate. First, the surface of the FTO substrate (7–8 Ω sq^−1^) was alternatively etched to separate the anode and cathode of each cell, as shown in Figure 1. Briefly, a 1.0 × 1.0 cm FTO substrate was partitioned vertically into four equal compartments using a simple wet etching procedure. Then, alternative etching was carried out horizontally on the top and bottom of the FTO substrate. As shown in Figure 1, NiO film was deposited on the cleaned, patterned FTO substrate at room temperature (RT) by spin coating using a 20 mg/mL NiO-DI water solution at 2000 rpm for 1 min. The thickness of the NiO layer on the FTO substrate was 55 nm, as calculated from its FE-SEM cross-sectional image. Next, the DMF:DMSO (8:2) solvent was spin-coated on the surface of the NiO layer to improve surface smoothness and hole extraction. Then, the as-prepared MAPbI_3_ solution (PbI_2_ and CH_3_NH_3_I in a 1:1 molar ratio at a concentration of 1.1 M in a mixture of DMF and DMSO (8:2)) was spin-coated on the FTO/NiO substrate at a rotation speed of 1000 rpm for 5 s and 5000 rpm for 30 s. Then, PCBM (an electron transporting material) in anhydrous chlorobenzene (20 mg/mL) was spin-coated on the MAPbI_3_ film. The substrate was then heated to 100 °C and held at this temperature for 10 min, and a very thin layer of bathocuprine in IPA (0.5 mg/mL) was deposited on the surface of the PCBM layer by spin-coating. Finally, 80 nm thick silver electrodes were deposited by thermal evaporation through a specialized shadow mask.

### 2.3. Characterizations

The surface morphologies of the LiFePO_4_-Li_4_Ti_5_O_12_ powders were examined using FE-SEM (SU-70 Hitachi High-Tech Corporation, Fukuoka, Japan). The accelerating voltage for the FE-SEM measurements was 5 kV.

The structural properties of the LiFePO_4_ and Li_4_Ti_5_O_12_ powders were analyzed using XRD (XRD-2500 Riguku, Japan) with Cu-k_α_ radiation. The XRD patterns of the powders were obtained over a 2θ range of 20°–70° with a step size of 0.05°and a scan rate of 1.0°/min.

The electrochemical properties of the LiFePO_4_-Li and Li_4_Ti_5_O_12_-Li half cells and LiFePO_4_-Li_4_Ti_5_O_12_ full cell were evaluated using a WonATech battery tester. Galvanostatic charge-discharge tests of the LiFePO_4_-Li_4_Ti_5_O_12_ full cell were carried out over the potential range of 1.0–2.5 V. In addition, the LiFePO_4_-Li_4_Ti_5_O_12_ full cell was evaluated at various C-rates (0.1, 0.2, 0.5, 1, and 2 C). The cycle retention of the LiFePO_4_-Li_4_Ti_5_O_12_ full cell after 100 cycles was also examined. 

## 3. Results

**Figure 2:**
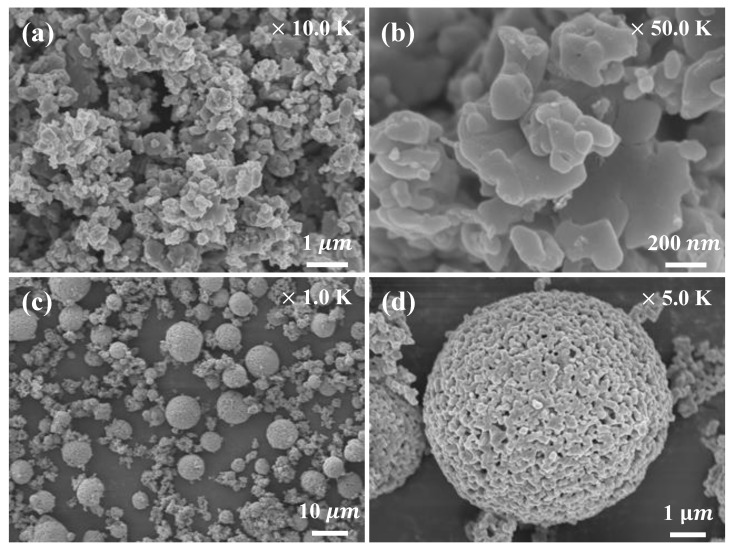
FESEM images of (**a**) and (**b**) LiFePO_4_, (**c**) and (**d**) Li_4_Ti_5_O_12_ powders.

FE-SEM images of the LFP powder used as the cathode material are shown in Figure 2a,b. The FE-SEM images show that the cathode material consisted of approximately spherical particles 0.1–1.0 µm in size. FE-SEM images of the LTO powder (the anode material) are shown in Figure 2c,d. The anode material consisted of fine primary particles and larger secondary particles formed by agglomeration of the primary particles. Figure 2d shows porous spherical particles approximately 10 µm in size. The porous, spherical secondary particles provided an ideal network for liquid electrolyte penetration and transport of lithium ions [21]. 

**Figure 3:**
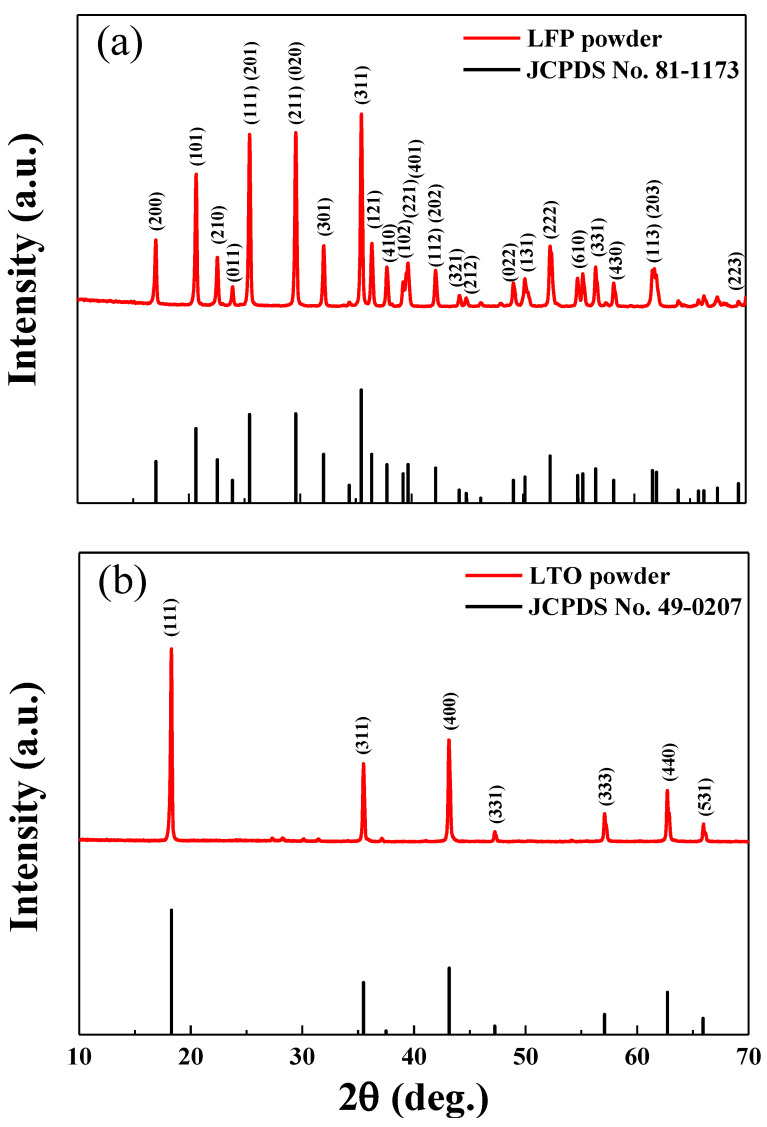
XRD patterns of (**a**) LiFePO_4_ powder and JCPDS card No. 81-1173 and (**b**) Li_4_Ti_5_O_12_ powder and JCPDS card No. 49-0207.

The XRD pattern of the LFP powder used to prepare the cathode is shown in Figure 3a. The XRD peaks of the LFP powders corresponded to the JCPDS data for LFP. The XRD pattern of the LTO powder (the anode material) is shown in Figure 3b. The XRD peaks of the LTO powder matched well with the JCPDS data for LTO. The XRD results confirmed successful synthesis of the LFP and LTP powders, without significant contamination.

**Figure 4:**
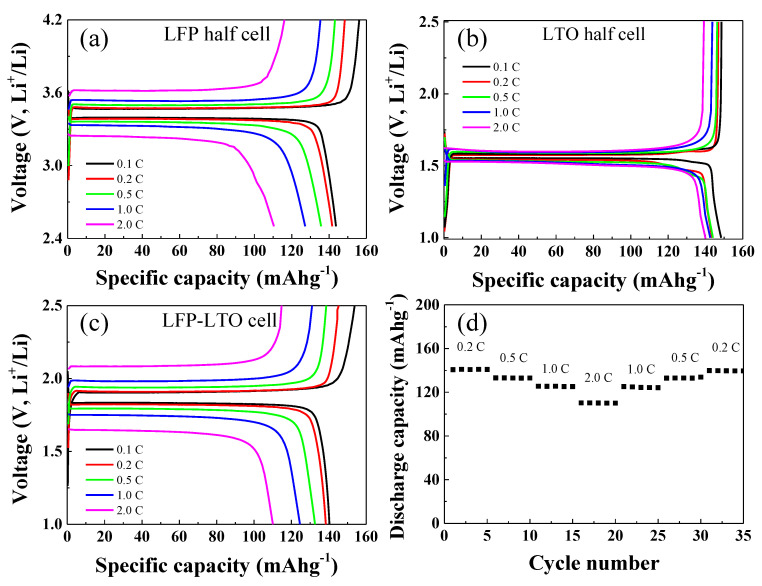
Electrochemical properties of the (**a**) LFP-Li, (**b**) LTO-Li, and (**c**) LFP-LTO cells and (**d**) rate capability of the LFP-LTO cell measured at various C-rates.

The LFP and LTO electrodes were galvanostatically tested in half-cell configurations paired with Li metal as the counter electrode. The results are shown in Figure 4. The galvanostatic measurements of the LFP-Li half-cell were carried out over the potential range of 2.5–4.2 V, while those for the LTO-Li cell were carried out over the potential range of 1.0–2.5 V. The galvanostatic charge-discharge measurements of both half-cells were carried out at C-rates of 0.1, 0.2, 0.5, 1, and 2 C. As shown in Figure 4a, the LFP-Li cell showed discharge capacities of about 143, 141, 138, 124, and 111 mAhg^−1^ at C-rates of 0.1, 0.2, 0.5, 1, and 2C, respectively. The discharge capacities of the LTO-Li cell were about 150, 145, 145, 143, and 143 mAhg^−1^ at C-rates of 0.1, 0.2, 0.5, 1, and 2C, respectively (Figure 4b). The charge/discharge capacities and capacity retention of the LFP-LTO full cell are shown in Figure 4c,d, respectively. The galvanostatic tests of the LFP-LTO full cell were carried out over the voltage range of 1.0–2.5 V. As shown in Figure 4c, the discharge capacity of the LTO-Li cell was about 141, 140, 132, 125, and 110 mAhg^−1^ at 0.1, 0.2, 0.5, 1, and 2C, respectively. The capacity retention test of the LFP-LTO cell was carried out by continuously charging and discharging, five times each, as shown in Figure 4d. The LFP-LTO full cell exhibited stable cycling performance at various C-rates. 

**Figure 5:**
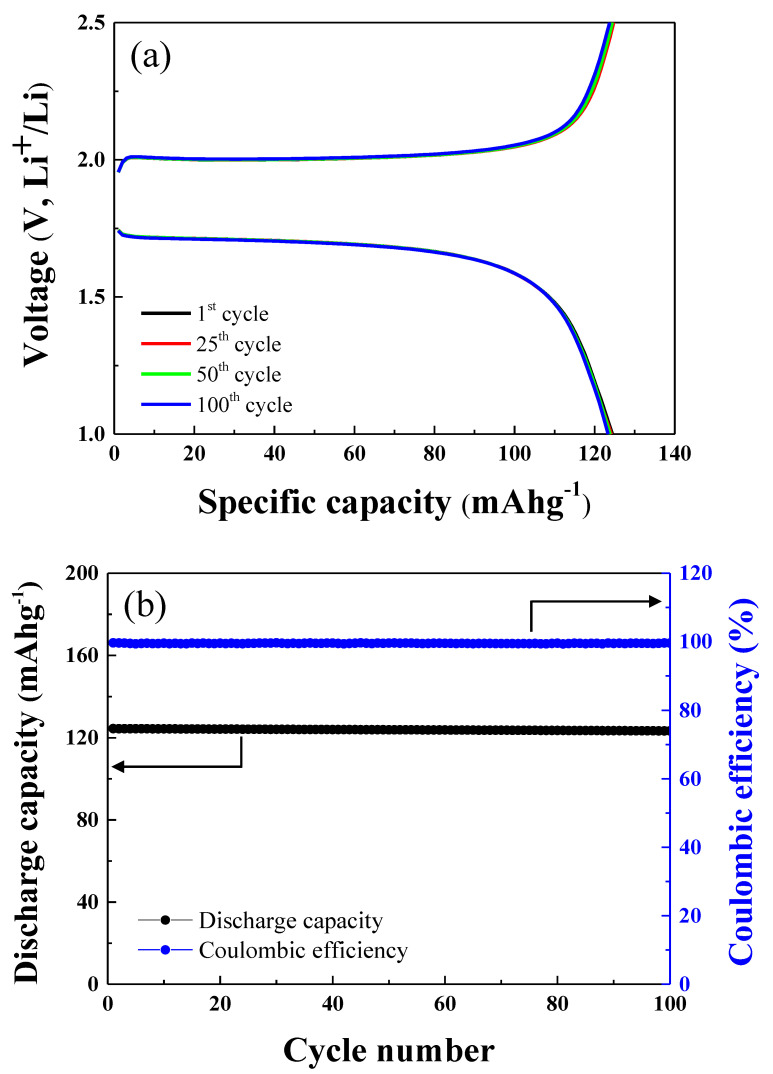
(**a**) Cycle performance of the LFP-LTO full cell at 1.0 C in the voltage range from 1.0 to 2.5 V for 100 cycles. (**b**) Discharge cycle retention and Coulombic efficiency for 100 cycles.

The cycle tests of the LFP-LTO full cell were carried out over 100 cycles at 1.0 C, as shown in Figure 5a. The charge and discharge capacity of the LFP-LTO cell after the 1st, 25th, 50th, and 100th cycles are shown in Figure 5a. The discharge capacity of the cell after the 1st, 25th, 50th, and 100th cycles was 124.42, 124.14, 123.85, and 123.33 mAhg^−1^, respectively. The discharge capacity and Coulombic efficiency of the LFP-LTO full cell after 100 cycles at 1 C are shown in Figure 5b. The LFP-LTO full cell showed 99.3% cycle retention after 100 cycles. It should be noted that the LFP-LTO full cell had an excellent cycle life, without significant degradation of discharge capacity. In addition, the full cell showed excellent Coulombic efficiency for 100 cycles, as shown in Figure 5b. The Coulombic efficiency of the LFP-LTP cell after the 1st, 25th, 50th, and 100th cycles was 99.64%, 99.59%, 99.55%, and 99.56%, respectively. The Coulombic efficiency remained the same for 100 cycles. This suggests that the LFP-LTO cell will display stable operation and a long cycle life.

**Figure 6:**
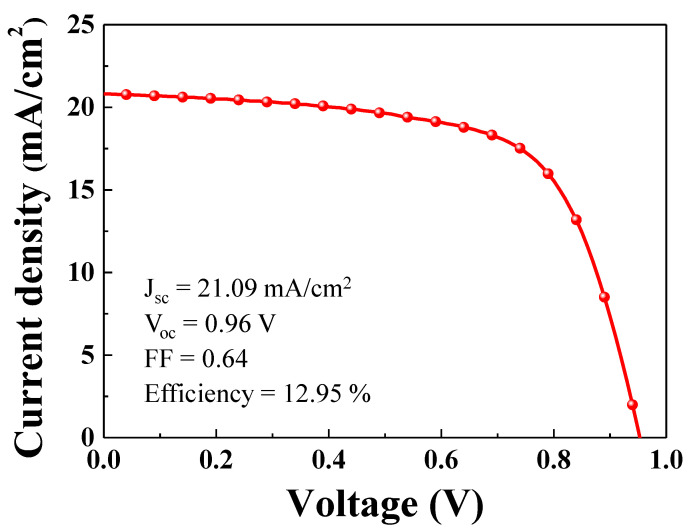
J-V curve of the single PSC.

We fabricated a PSC with an FTO/NiO (200 nm) structure using ETL/CH_3_NH_3_PbI_3_ (perovskite, 250 nm)/phenyl-C61-butyric acid methyl ester (PC61BM, 100 nm) as an HTL/Ag (100 nm). Before integration of the self-charging system, the performance of a single PSC was investigated. Figure 6 shows the current density-voltage (J-V) characteristics of a single PSC. The PSC showed a short circuit photocurrent density of 21.09 mA/cm^2^, which matched well with the values calculated from the incident photo-to-current efficiency spectrum. The open circuit voltage and fill factor were 0.96 V and 0.64, respectively. The single PSC showed a power conversion efficiency of 12.95%. 

**Figure 7:**
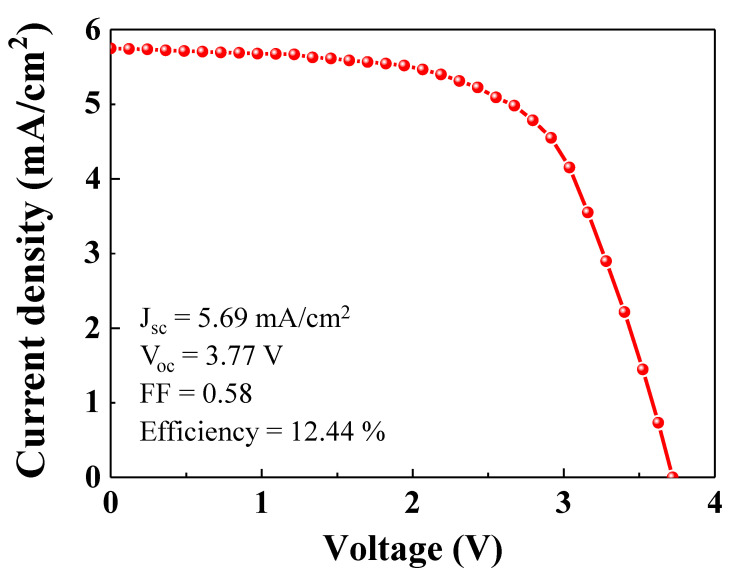
J-V curve for the integrated PSC unit, comprising four single PSCs connected in series.

Figure 7 shows the photocurrent-voltage curve (*J*-*V*) for four single PSCs in series. As shown in Figure 5a, in order to charge the LIB, the input voltage must exceed the plateau voltage (>~2.0 V). As shown in Figure 6, the open circuit voltage and fill factor of the single PSC were 0.96 V and 0.64, respectively. Given the fill factor of the single PSC, the operating voltage is ~0.6 V. Therefore, in order to get enough voltage from the PSC to charge the LIB, at least four PSCs must be placed in series, which gives an operating voltage of over 2.0 V. As shown in Figure 7, the operating voltage of the four PSCs in series was ~2.4 V, which was sufficient to charge the LIB. As shown in Figure 7, the short circuit photocurrent density for the integrated PSC device was 5.69 mA/cm^2^. The open circuit voltage and fill factor were 3.77 V and 0.58, respectively. The fill factor of the integrated PSC device was slightly lower than that of the single PSC. The integrated PSC device showed a power conversion efficiency of 12.44%.

**Figure 8:**
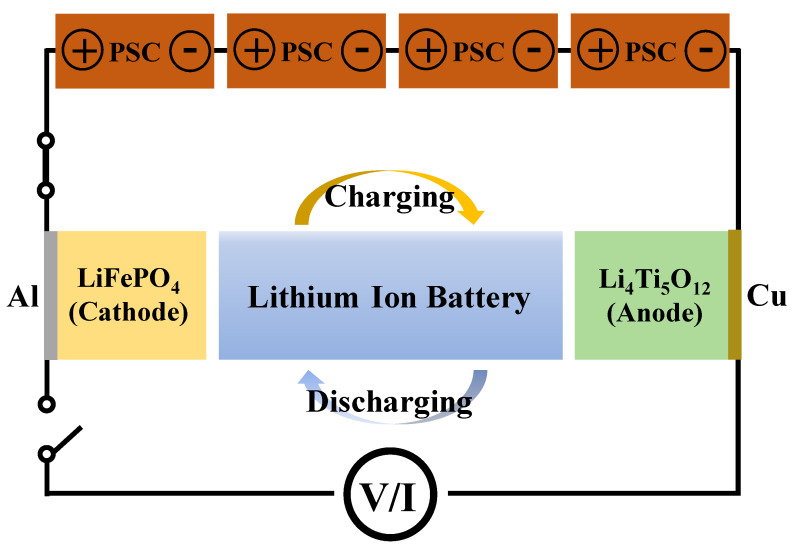
Schematic of the PSCs-lithium-ion batteries (LIB) coupling device.

Figure 8 shows the schematic of the PSCs-LIB integrated system. As mentioned earlier, an LFP cathode and LTO anode were used to assemble the LIB. In order to ensure a sufficiently high operating voltage for the self-charging system, PSCs-LIB, a PSC pack was designed by connecting four single PSCs together in series. In this study, a solar simulator was used for the PSC photocurrent. As shown in Figure 8, the photogenerated free hole and electrons from the PSCs can flow into the LFP cathode and LTO anode, respectively, of the LIB. Photocurrent flow from the PSCs to the closed-circuit LIB charge the LIB. The energy conversion and storage efficiencies of the PSCs-LIB integrated device were 9.25%. In addition, the energy storage efficiency of the PSC for photo-charging of the LIB was 74.3%. Notably, a device made of high-efficiency PSCs and an LIB is quite efficient in terms of energy storage.

**Figure 9:**
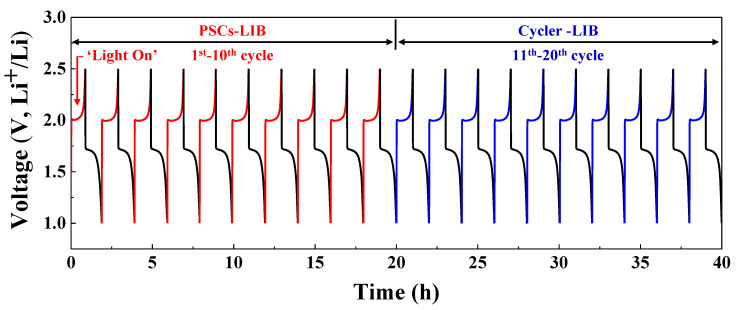
Phtotocharging and discharging diagram of the LFP-LTO cell using PSCs (from cycles 1 to 10) and direct current (DC) charging and discharging using the cycler (from cycles 11 to 20).

Figure 9 shows the photocharging of the integrated PSC device and its galvanostatic discharge voltage profile during ten cycles in the potential voltage range of 1.0–2.5 V. During the next ten cycles, the integrated PSC device showed charge/discharge behavior (tested using a battery cycle tester) that was the same as that of the LFP-LTO cell over the potential range of 1.0–2.5 V. The PSCs-LIB device and the LIB cell without PSCs (charged with the battery cycler) showed similar charge/discharge voltage profiles. Hence, photocharging (self-charging) of LIBs using integrated PSCs connected in series is an efficient approach to direct current (DC) charging (comparable to the use of a battery cycler). Therefore, a self-charging system using the PSCs-LIB is a promising method of supplying power to portable electronic devices, EVs, and many other electronic systems.

**Figure 10:**
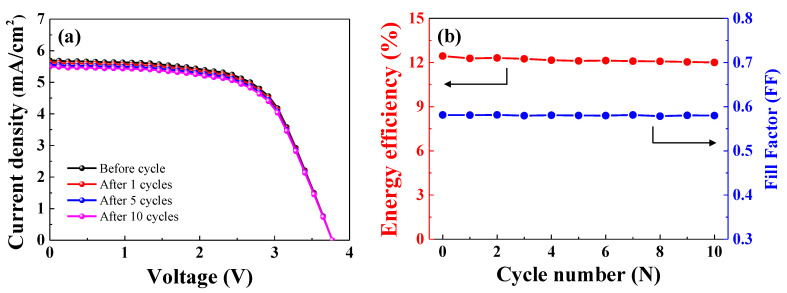
J-V curve (**a**) and energy efficiency and fill factor (**b**) of the PCSs connected to the LIB before and after various cycles.

As shown in Figure 9, the PSCs-LIB device was subjected to 10 cycles of photo-charging and glavanostatic discharging. Figure 10a shows the J-V curve of the PSCs connected to the LIB after various cycles (1, 5, and 10 cycles). Before the cycle test, the current density of the PSCs connected to the LIB was 5.69 mA. As cycle number increased, the current density of the PSCs connected to the LIB decreased slightly. The current density during the 1st, 5th, and 10th cycle was 5.62, 5.54, and 5.49 mA, respectively. The open circuit voltage of the PSCs did not change significantly over the course of 10 cycles. The fill factor and energy efficiency of the PSCs before and after each of 10 cycles are shown in Figure 10b. Before the cycle test, the conversion efficiency of the PSCs was 12.44%. As cycle number increased, the energy conversion efficiency slightly decreased. The energy conversion efficiency of the PSCs connected to the LIB during the 1st, 5th, and 10th cycle was 12.28%, 12.11%, and 12.00%, respectively. The fill factor did not significantly differ before and after the cycles. The energy conversion efficiency of the PSCs connected to the LIB during the 10th cycle was 96.46% of the efficiency before the cycling test. Thus, high-efficiency PSCs in series connected to a LIB are promising as a power supply.

## 4. Conclusions

In summary, we fabricated a self-charging LIB system with four single PSCs connected in series. To ensure highly efficient self-charging of the LIB with LFP as the cathode and LTO as the anode, a pack of four single PSCs were connected in series. A single PSC showed a fill factor and power conversion efficiency of 0.64 and 12.95%, respectively. Although the four single PSCs were connected in series, the integrated device showed a fill factor and power conversion efficiency of 0.58 and 12.44%, respectively, which are slightly lower than those of a single PSC. Photocharging of the LIB using the integrated PSC device was as effective as DC charging using a battery cycler. Therefore, the use of series-connected PSCs is an efficient approach to photocharging of LIBs for applications in electronic devices.
